# Which growth parameters can affect mortality in cerebral palsy?

**DOI:** 10.1371/journal.pone.0218320

**Published:** 2019-06-14

**Authors:** Hyun Jung Kim, Tae Uk Kang, Kyu Yong Park, Jihyun Kim, Hyeong Sik Ahn, Shin-Young Yim

**Affiliations:** 1 Department of Preventive Medicine, College of Medicine, Korea University, Seoul, Republic of Korea; 2 Health Insurance Policy Research Institute, National Health Insurance Service, Wonju, Republic of Korea; 3 Department of Physical Medicine and Rehabilitation, Ajou University School of Medicine, Suwon, Republic of Korea; 4 Research Institute, National Cancer Center, Goyang, Republic of Korea; University of Bern, SWITZERLAND

## Abstract

**Background:**

The objective of this study was to identify growth parameters that can affect mortality of cerebral palsy (CP).

**Method:**

This was a birth cohort study based on the National Health Screening Program for Infants and Children database along with the National Health Insurance Service, which were linked using a personal identifier number. The birth cohort consisted of 2 191 956 subjects, representing 93.5% of live births from 2007–2011, with maximal 10-year follow-up (range, 5–10 years) until October 2016. Subjects with CP were identified. Growth parameters in terms of birth weight, underweight (weight-for-age below the 3rd percentile), rate of body weight gain were collected, along with all-cause mortality after the age of 1 year.

**Result:**

Prevalence of CP was 2.0 per 1000 live births (95% CI, 1.94–2.06). All-cause mortality after the age of 1 year was 0.09 deaths/1000 person-years (95% CI, 0.08–0.09) in the general population (GP) and 2.85 deaths/1000 person-years (95% CI, 2.32–3.50) in subjects with CP during the follow-up. Therefore, the incidence rate ratio for all-cause mortality was 32.15 (95% CI, 25.72–39.76) in subjects with CP compared to GP. Presence of underweight was significantly associated with higher mortality in both subjects with CP and GP, where the adjusted hazard ratio of death was 2.60 (95% CI, 1.93–3.50) at the age of 18–24 months, 3.12 at 30–36 months, 4.37 at 42–48 months, 5.12 at 54–60 months, and 4.17 at 66–71 months. Birth weight did not affect mortality in both subjects with CP and GP after the age of 1 year (*p* > 0.05).

**Conclusion:**

While subjects with CP shows higher mortality, underweight is an important growth parameter that affects all-cause mortality of both subjects with CP and GP. This study urges increased awareness that subjects with CP who are underweight require special care.

## Introduction

Cerebral palsy (CP) describes a group of permanent disorders affecting the development of movement and posture, causing activity limitations that are attributed to non-progressive disturbances which occur in the developing fetal or infant brain [1,2]. CP is the most common cause of motor disability in childhood [3]. CP has an adverse influence on many aspects of growth, survival, and the daily functioning of subjects. It also imposes demands on health, social, and educational services [4].

Understanding the prevalence and clinical outcomes of subjects with CP (SCP), such as longitudinal growth and mortality, is an essential step in the management of SCP. While there are many studies on growth and mortality of SCP, most studies were based on CP cohorts and followed only SCP [5]. No studies simultaneously followed up SCP and general population (GP). Therefore, we do not know the real difference of the longitudinal growth and mortality between SCP and GP. No studies compared growth parameters such as birth weight (BW), weight-for-age, and the rate of body weight gain all together between SCP and GP. No report analyzed growth parameters as mortality risk factors in both SCP and GP, either.

The prevalence and clinical outcomes of CP are influenced by several factors related to the socioeconomic status of each society, such as the availability of medical services, the level of medical knowledge, and technology. The majority of data on prevalence, growth parameters [3,6–11] and mortality of CP [12–18] are based on CP cohorts from European countries, Australia, and the United States of America. There is very little Asian data on prevalence, BW, weight-for-age, the rate of body weight gain, and mortality of SCP. The objective of this study was to identify growth parameters that can most affect mortality of CP.

## Materials and methods

We conducted a population-based study of Korean children born from January 1^st^, 2007–December 31^st^, 2011. We observed the Record Statement (http://record-statement.org, S1 Checklist). This study was approved as an exempted study for Ajou University Hospital Institutional Review Board (MED-EXP-17-174).

### Data source

Data were retrieved from two South Korean databases that are managed by the National Health Insurance Service (NHIS): (1) the National Health Screening Program for Infants and Children (NHSIC) database, which records screening data of the growth and development of all children [19]; (2) the NHIS database, which covers the entire population and includes comprehensive health claims data [20]. The NHSIC database and the NHIS database are linked using a personal identifier number.

The Korean government launched the NHSIC program in November 2007 as a population surveillance system, with the goal of improving the health and well-being of children. The NHSIC screening is performed at the age of 4–6 months (1st), 9–12 months (2nd), 18–24 months (3rd), 30–36 months (4th), 42–48 months (5th), 54–60 months (6th), and 66–71 months (7th). While the NHSIC screening is not mandatory but optional, children can participate in the NHSIC program up to maximal 7 times, from the age of 4–71 months. Physicians conduct anthropometric measurements as well as history taking, physical examination, screening for visual acuity, and health education. NHSIC data is submitted to the NHIS by participating clinics [19].

The NHIS database of South Korea is a repository of claims data collected in the process of reimbursing healthcare providers. Under the universal coverage system, having fee-for-services covering all citizens in South Korea, the NHIS database contains comprehensive and rich information pertaining to healthcare services such as treatments, pharmaceuticals, procedures, and diagnoses for almost 50 million beneficiaries. The NHIS database includes data on all children, regardless of presence of disability.

### The 2007 to 2011 birth cohort

**Fig 1:**
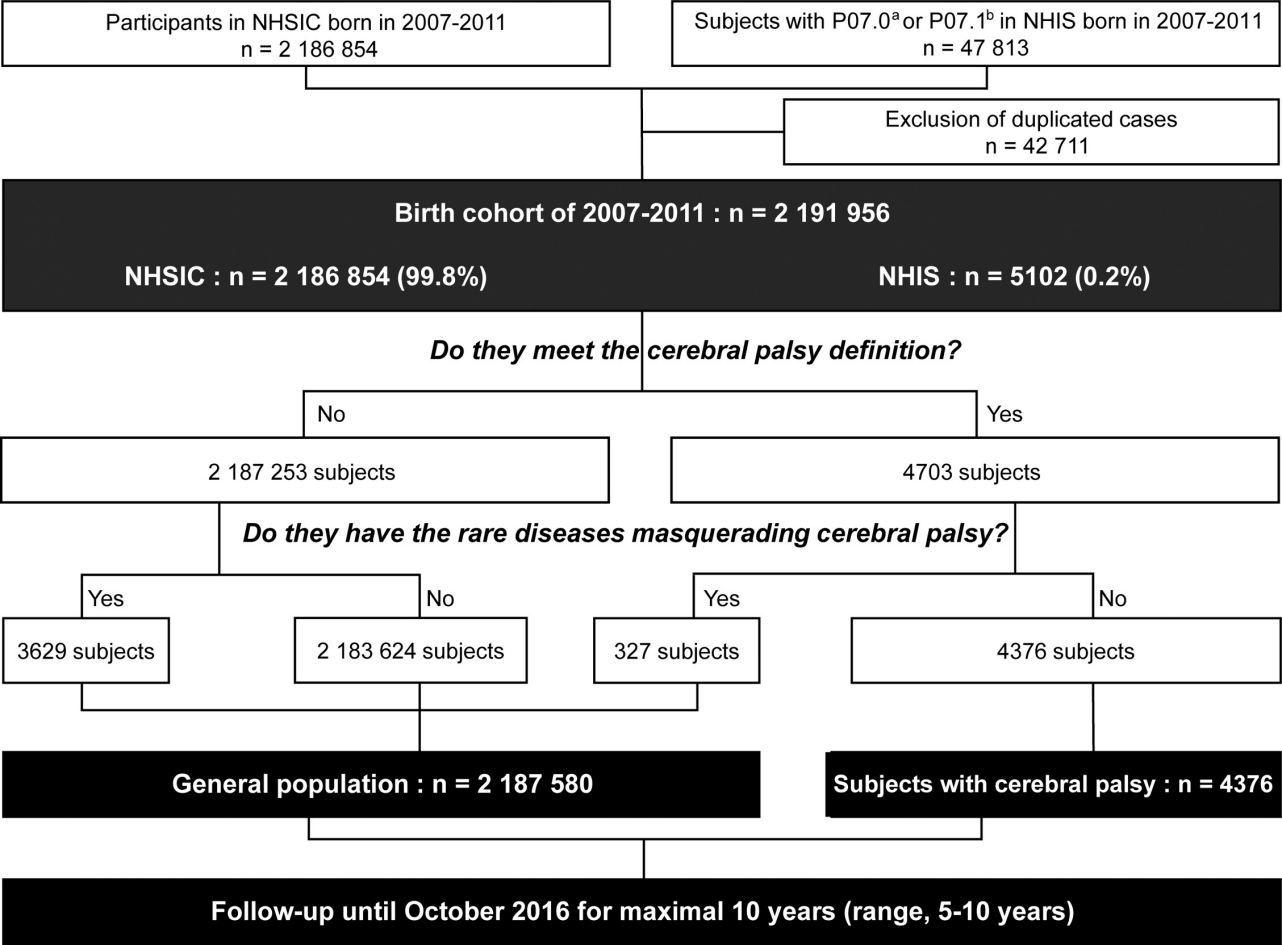
The flow diagram of development of the 2007 to 2011 birth cohort. NHSIC, the National Health Screening Program for Infants and Children; P07.0, the code of BW <1000 g by the Korean Classification of Diseases; P07.1, the code of 1000 g ≤ BW <2500 g in the Korean Classification of Diseases; NHIS, the National Health Insurance Service.

We enrolled subjects born from 2007–2011 who had participated in the NHSIC program at least one time. Since the NHSIC is a population surveillance program for children from the age of 4 months, infants who died before this age were unable to participate in the program. While the NHSIC screening is optional, participation in the NHSIC screening was relatively low in children who had already received in-depth medical attention for any particular reason, including low BW and prematurity. We tried to identify as many children who had not participated in the NHSIC as possible, especially children with low BW. We identified subjects with low BW who had not participated in the NHSIC, using the NHIS database. The NHIS database applies the Korean Classification of Diseases, which is a modified version of the International Classification of Diseases, 10^th^ edition. Subjects with low BW were identified through the Korean Classification of Diseases codes P07.0 and P07.1, which indicate BW <1000 g and 1000 g ≤ BW <2500 g, respectively. After excluding 42 711 duplicated cases between the NHSIC and NHIS database, the 2007 to 2011 birth cohort was finally developed. Fig 1 shows the flow diagram of development of the birth cohort, which was followed up until October 2016 for maximal 10 years (range, 5–10 years).

### Identification of SCP

Subjects in the birth cohort were classified as either GP or SCP. The GP represented all subjects except SCP. In the birth cohort, we identified SCP using the NHIS database, the Disability Registry (DR), and the Rare Intractable Disease database.

The DR database, which is a registry of persons with disabilities, requires an evaluation executed by a physician specialized in the related medical field, thus ensuring diagnostic accuracy. While individuals with CP are registered under the category of disability associated with brain lesions, this category includes not only CP, but also traumatic brain injury, stroke, and other brain lesions. The DR database does not provide a specific diagnostic category for CP and there are SCP who did not register in the DR. Therefore, we first identified subjects with the diagnosis code of CP from the NHIS database (G80 of Korean Classification of Diseases code) at least once. Since the G80 code does not always represent actual SCP, 16 operational definitions of CP were made for subjects with G80 by combining one or more of the 4 conditions (S1 Table). We finally chose the following definition out of 16 operational definitions, where subjects were identified as having CP when they had the G80 code and met one or more of the following 4 conditions: (1) having both one or more admissions and 5 or more visits to a clinic as an out-patient with G80; (2) having 10 or more visits as an out-patient within 3 consecutive years with G80; (3) having a disability registration with brain lesions, one or more admissions, and 2 or more visits as an out-patient with G80; or (4) having a disability registration with brain lesions and 5 or more visits to a clinic as an out-patient with G80.

We also excluded subjects who had known conditions masquerading as CP from SCP, using the codes of the Rare Intractable Disease database (Fig 1, S2 Table), which is a registry for rare diseases [21].

### Data collection

Data cleaning was done before data collection, removing extra spaces, treating all blank cells and removing duplicates. Supplemental information such as the study protocol, raw data, or programming code is available by contacting the authors.

Data on gender, birth date, and the age at diagnosis of CP were collected from the NHIS database. The age at each NHSIC screening was collected from the NHSIC database. The number of live births from 2007–2011 in South Korea was collected from Statistics Korea (http://kostat.go.kr). All deaths after the age of 1 year were captured in the NHIS database as of October 2016. Since all deaths were reported in the NHIS database, if no death was recorded in the NHIS database as of October 2016, there would be little possibility that the individual was dead as of October 2016.

Growth parameters were collected in terms of BW, the follow-up body weight, the follow-up weight-for-age percentile and the rate of body weight gain after the age of 1 year. BW was collected from the NHSIC database for subjects who participated in the NHSIC program, where BW data was recorded based on a questionnaire filled out by parents. In cases where BW was not available from the NHSIC or subjects who did not participate in the NHSIC, BW was identified from the NHIS database where BW only could be stratified as 3 categories such BW <1000 g, 1000 g ≤ BW <2500 g and BW ≥2500 g by the Korean Classification of Diseases codes such as P07.0 (BW <1000 g) or P07.1 (1000 g ≤ BW <2500 g).

The follow-up body weight data and the follow-up weight-for-age percentile data were collected from the NHSIC database after the age of 1 year, which were measured and inputted by a physician at each time of the NHSIC screenings. We collected the data after the age of 1 year, including the 5 NHSIC screenings from the 3rd (18–24 months) to the 7th (66–71 months). Since not all children in the current birth cohort participated in all NHSIC screenings, we could get the data on the follow-up body weight and the follow-up weight-for-age percentile data only for children who participated in the NHSIC screenings. The total number of participation in the NHSIC screenings varies for each child.

As a definition of abnormal growth, the 2006 World Health Organization (WHO) recommends cutoff values of ±2 standard deviations, which correspond to the 2.3rd and 97.7th percentiles [22]. In the current study, the follow-up weight-for-age percentile data were provided as an integer without any decimal places. We defined underweight as weight-for-age below the 3rd percentile [23].

The rate of body weight gain after the age of 1 year was calculated as follows: body weight at the time of the NHSIC screening-BW/age of the subjects at the time of the NHSIC screening (g/day).

### Statistical analysis

CP prevalence was calculated by dividing the number of SCP by the total number of live births. Gender- and BW-specific prevalence of CP were also obtained. All-cause mortality with maximal 10-year follow-up until October, 2016 was calculated as follows: number of deaths/1000 person-years. Incidence rate ratio for all-cause mortality was also calculated as follows: mortality of SCP/ mortality of subjects in the GP.

Independent t-tests were used to compare BW, the follow-up body weight, the rate of body weight gain, and the age at each NHSIC screening between GP and SCP. The chi square test was used to compare the number of subjects who are underweight between GP and SCP.

The Cox proportional hazard regression analysis was used to examine the relation between all-cause mortality until October 2016 and explanatory factors such as the presence of CP, gender, birth year, BW, and the presence of underweight, in terms of the hazard ratio (HR) of death with 95% confidence interval (CI). Since the presence of underweight could be dependent on the rate of body weight gain, we excluded the rate of body weight gain in Cox proportional hazard regression analysis. We tested the proportional hazards assumption based on the scaled Schoenfeld residuals. The global test was non-significant for all 5 NHSIC screenings. Therefore, we validated the proportional hazards assumption. The Cox proportional hazard regression analysis was individually done for each NHSIC screening.

All significance levels were 2-sided, with *p-*value of less than 0.05 indicating statistical significance. Stata/MP2 (version 13.1; StataCorp, College Station, TX, USA) was used in the analyses.

## Results

### Characteristics of the 2007 to 2011 birth cohort

The 2007 to 2011 birth cohort consisted of 2 191 956 subjects (Fig 1, S3 Table). The 99.8% of the subjects in this study came from the NHSIC and 0.2% came from the NHIS. The birth cohort represents 93.5% of the total live births from 2007–2011 in South Korea. Since it was not possible to generate exact data on ethnicity for the current cohort, we estimated this from the Korean Statistical Information Service (http://kosis.kr). As of 2016, the number of children under the age of 9 in South Korea was 4 566 168. There were 24 237 immigrants and long term residents under the age of 9 from other countries in South Korea. Ethnically non-Korean children under the age of 9 were estimated to be 0.53% of the total number of children under the age of 9, since accurate numbers were not available.

### Prevalence of CP

Among the 2 191 956 birth cohort subjects, there were 3956 subjects that were identified as having conditions masquerading as CP (S2 Table). There were 76 subjects who had more than one condition, showing 4032 diagnoses. Finally, 4376 individuals were identified as having CP, with a boy-girl ratio of 1.4:1 (Table 1). Prevalence of CP was 2.0 per 1000 live births (95% CI, 1.94–2.06, Table 1). The age of CP diagnosis was 1.32 ± 1.13 years. Prevalence of CP was 2.2 per 1000 live births (95% CI, 2.16–2.34) for boys and 1.7 per 1000 live births (95% CI, 1.65–1.81) for girls, showing a higher prevalence of CP in boys (*p* < 0.001). The BW-specific prevalence of CP was 147.3 (95% CI, 135.83–159.65), 15.9 (95% CI, 15.18–16.70), and 1.1 (95% CI, 1.01–1.10) per 1000 live births for subjects with BW <1000 g, 1000 g ≤ BW <2500 g and BW ≥2500 g, respectively (Table 1).

**Table 1:** Prevalence^a^ of cerebral palsy (CP) in the 2007 to 2011 birth cohort by birth weight (BW).

Gender	Prevalence of subjects with CP (95% confidence interval, number of subjects with CP/number of live births)				
	Total subjects	BW <1000 g	1000 g ≤ BW <2500 g	BW ≥2500 g	Unknown BW
Boys	2.2 (2.16–2.34, 2540/1 129 127)	175.6 (157.54–195.34, 274/1560)	19.8 (19.77–21.04, 977/49 417)	1.2 (1.13–1.26, 1284/1 076 315)	2.7 (1.13–6.53, 5/1835)
Girls	1.7 (1.65–1.81, 1836/1 062 829)	123.4 (109.20–139.26, 228/1847)	12.5 (11.63–13.48, 700/55 908)	0.9 (0.85–0.96, 906/1 003 299)	(0.28–4.50, 2/1775)
Total	2.0 (1.94–2.06, 4376/2 191 956)	147.3 (135.83–159.65, 502/3407)	15.9 (15.18–16.70, 1677/105 325)	1.1 (1.01–1.10, 2190/2 079 614)	1.9 (0.92–4.06, 7/3610)

BW, birth weight

^a^Prevalence = number of CP/number of live births X 10^3^

Growth parameters in SCP

The age at the NHSIC screenings was compared between the subjects in the GP and SCP (S4 Table). There was no significant difference of the age at the NHSIC screenings between the GP and SCP, except the 3rd NHSIC screening (18–24 months). The age at the 3rd NHSIC screening was 10.18 days older in SCP than GP (*p* < 0.001).

**Fig 2:**
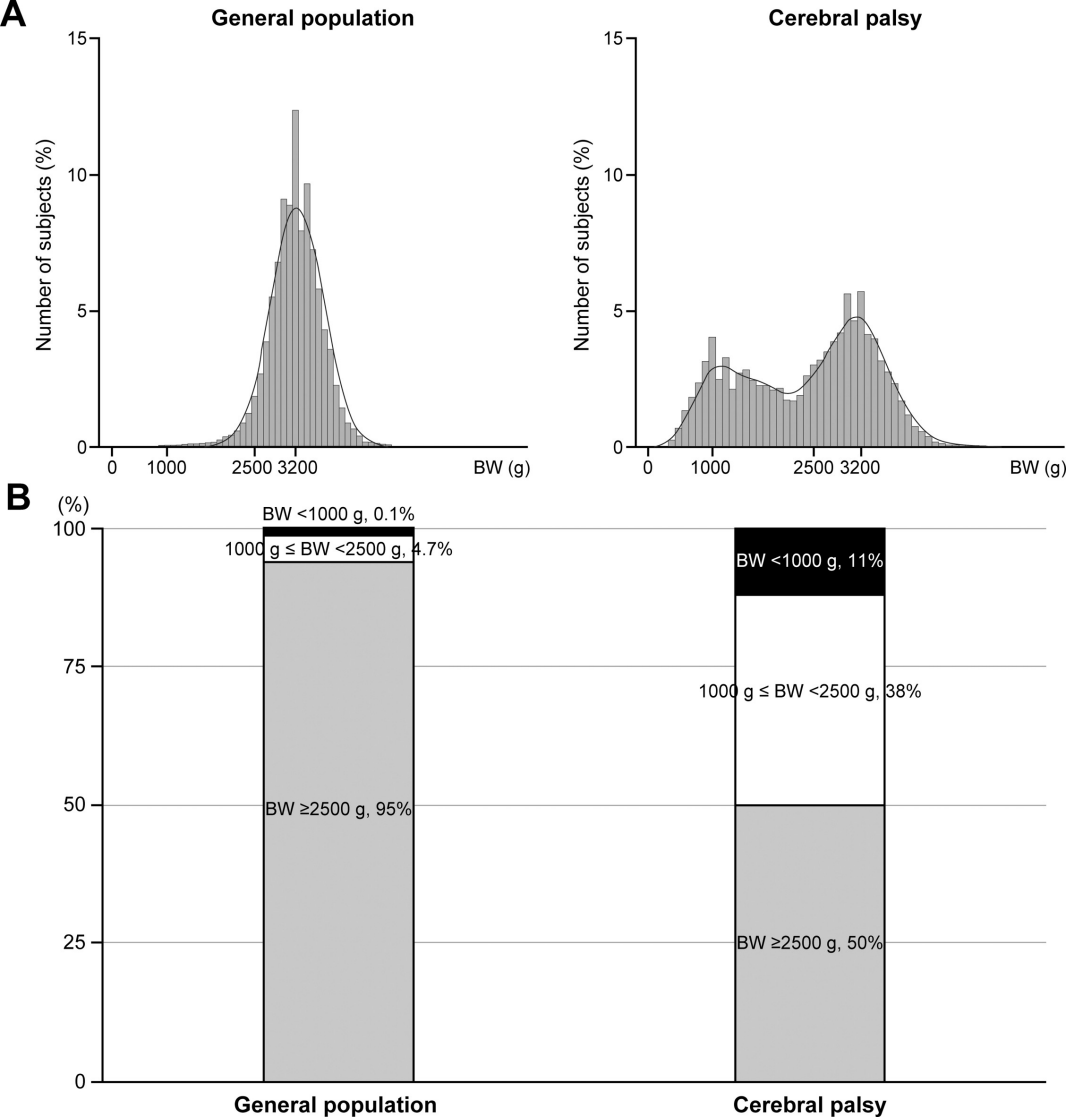
Comparison of birth weight (BW). (A) Distribution of subjects by BW. (B) Distribution of subjects by stratification of BW. Comparison was made between subjects in the general population and subjects with cerebral palsy.

Fig 2A shows the distribution of subjects by BW. The subjects in the GP exhibited a unimodal distribution of BW with one mode at 3200 g, while SCP showed a bimodal distribution of BW with 2 modes at 1000 g and 3200 g. An explanation for the bimodal distribution for CP is that there is a mixture between term infants and premature infants. The diagram of Fig 2B shows that subjects with BW <2500 g constituted 4.8% of the GP. Forty-nine percent of SCP had BW <2500 g, where 11% and 38% of SCP had BW <1000 g and 1000 g ≤ BW <2500 g, respectively.

**Fig 3:**
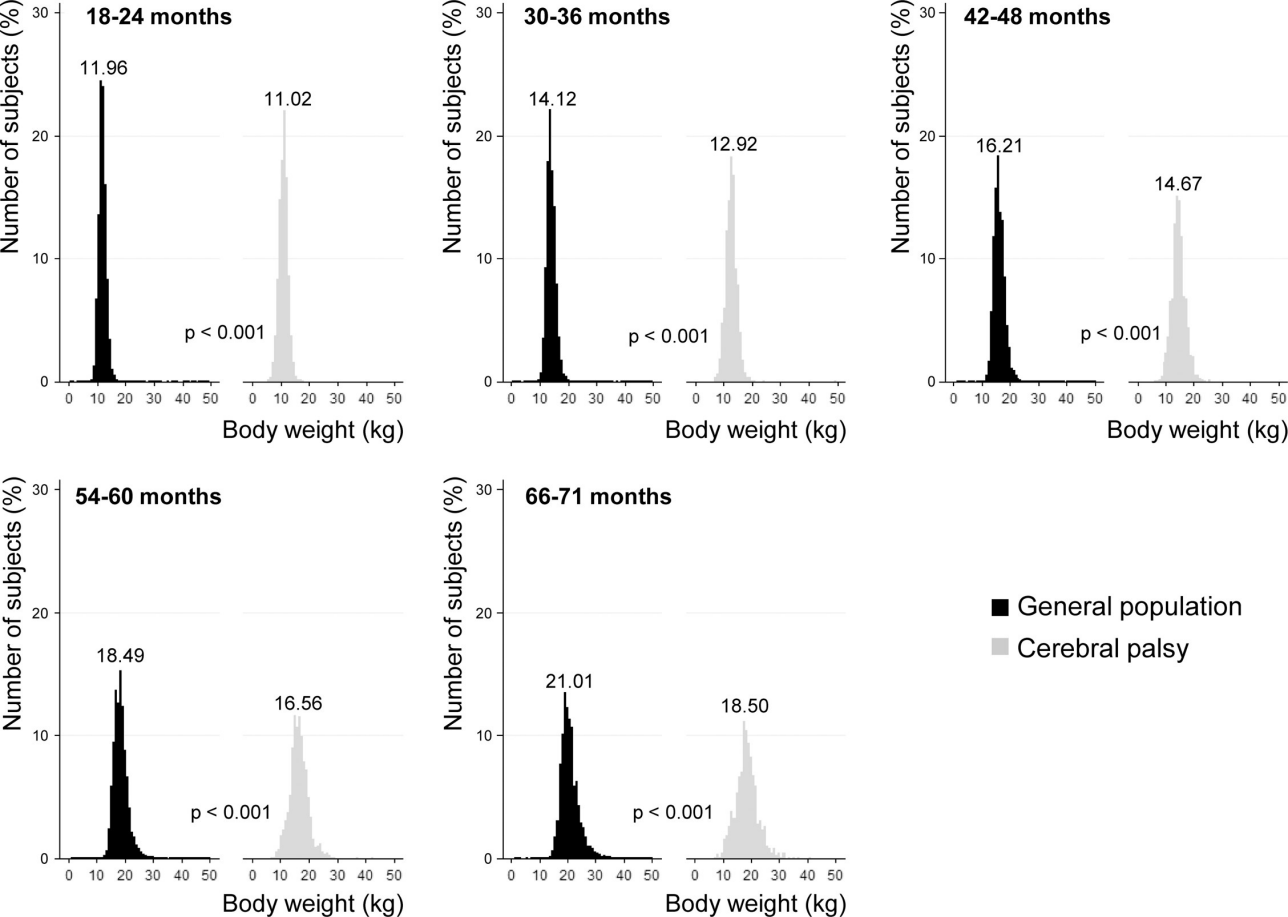
Comparison of follow-up body weight. Comparisons were made for follow-up body weight between subjects in the general population and subjects with cerebral palsy at each National Health Screening Program for Infants and Children. The number on each graph is the mean value of follow-up body weight.

Fig 3 shows the distribution of subjects by follow-up body weight at each NHSIC screening after the age of 1 year. While both the subjects in the GP and SCP showed similar unimodal distribution, their body weight increased as they grew older. The follow-up body weight was significantly lower in the SCP than the subjects in the GP at all NHSIC screenings after the age of 1 year (*p* < 0.001). The difference of the mean body weight between the subjects in the GP and SCP was 0.94 kg at the age of 18–24 months, 1.20 kg at the age of 30–36 months, 1.54 kg at the age of 42–48 months, 1.93 kg at the age of 54–60 months, and 2.51kg at the age of 66–71 months. It represents 7.86% (0.94 / 11.96 X 100 = 7.86) less body weight in SCP at the age of 18–24 months, 8.50% less body weight in SCP at the age of 30–36 months, 9.50% less body weight in SCP at the age of 42–48 months, 10.44% less body weight in SCP at the age of 54–60 months, and 11.95% less body weight in SCP at the age of 66–71 months, compared to the subjects in the GP. The difference of the body weight between the subjects in the GP and SCP became bigger as the children became older, from 7.86% less body weight at the age of 18–24 months to 11.95% at the age of 66–71 months in the SCP compared to the subjects in the GP.

**Fig 4:**
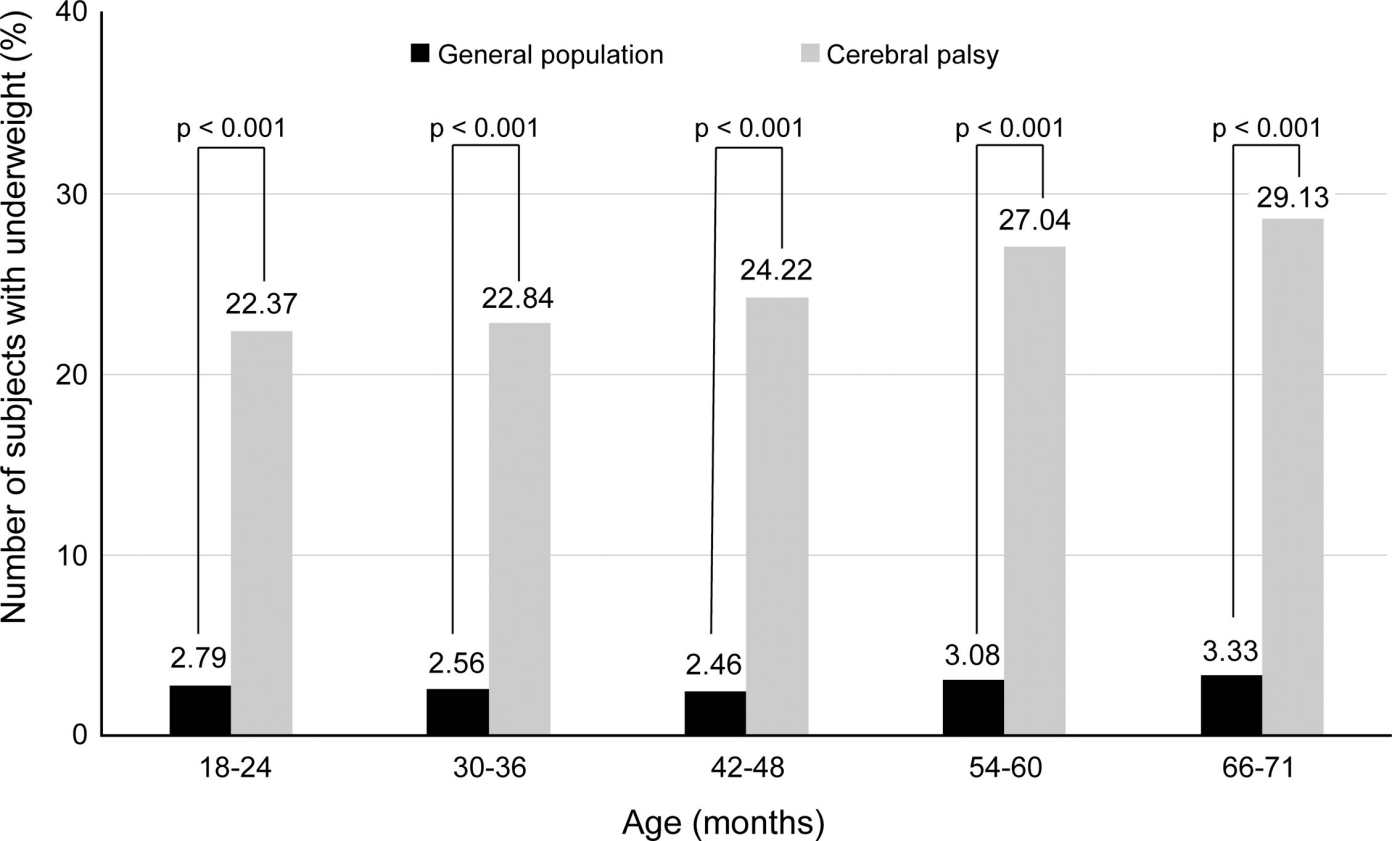
Comparison of underweight. Comparisons were made between the subjects in the general population and subjects with cerebral palsy at each National Health Screening Program for Infants and Children screening after the age of 1 year. The number on each bar is the mean value of the percentage of subjects who are underweight.

Fig 4 shows the frequency distribution of subjects who are underweight at each NHSIC screening after the age of 1 year. Underweight was found in 2.46%–3.33% of GP and 22.37%–29.13% of the SCP, indicating that underweight was significantly more common in SCP than in subjects in the GP at all NHSIC screenings after the age of 1 year (*p* < 0.001). The proportion of the subjects who are underweight became more as the children became older, from 22.37% at the age of 18–24 months to 29.14% at the age of 66–71 months in the SCP.

**Fig 5:**
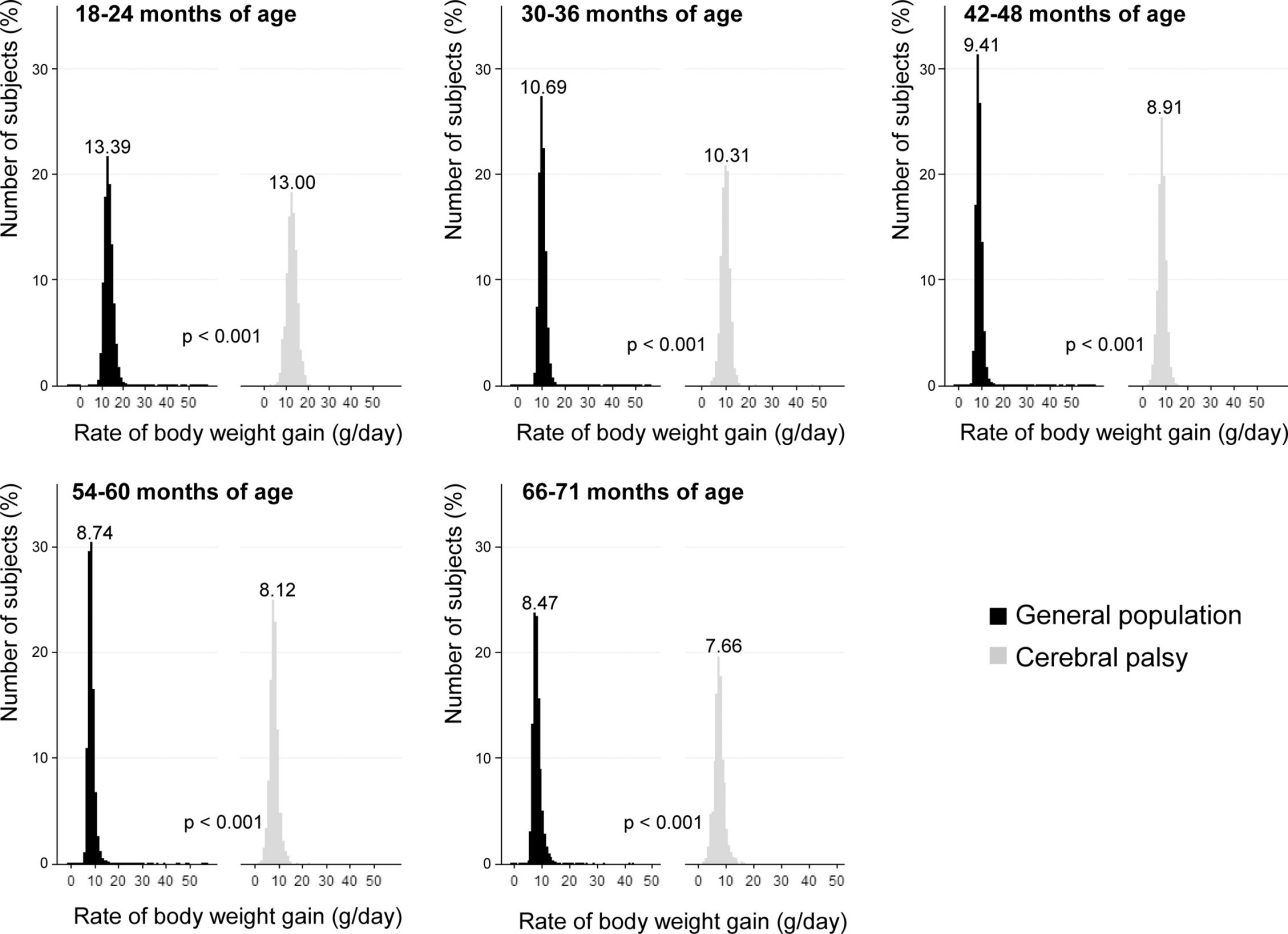
Comparison of the rate of body weight gain. Comparisons were made between the subjects in the general population and the subjects with cerebral palsy at each National Health Screening Program for Infants and Children. The number on each graph is the mean value of body weight for the subjects in the general population and subjects with cerebral palsy.

Fig 5 shows the distribution of subjects by the rate of body weight gain at each NHSIC screening after the age of 1 year. While both the subjects in the GP and SCP showed similar unimodal distribution, the rate of body weight gain decreased as they grew older in both groups. The rate of body weight gain was significantly slower in the SCP than the GP at all NHSIC screenings after the age of 1 year (*p <* 0.001). The difference of the mean rate of body weight gain between the subjects in the GP and SCP was 0.39 g/day at the age of 18–24 months, 0.38 g/day at the age of 30–36 months, 0.50 g/day at the age of 42–48 months, 0.62 g/day at the age of 54–60 months, and 0.81 g/day at the age of 66–71 months. As to the clinical meaning of these differences, it represents 2.91% (0.39 / 13.39 X 100 = 2.91%) less rate of body weight gain in SCP at the age of 18–24 months, 3.55% less rate of the body weight gain in SCP at the age of 30–36 months, 5.31% less rate of the body weight gain in SCP at the age of 42–48 months, 7.09% less rate of the body weight gain in SCP at the age of 54–60 months, and 9.56% less rate of the body weight gain in SCP at the age of 66–71 months, compared to the subjects in the GP. The difference of the mean rate of body weight gain between GP and SCP became bigger as the children became older, from 2.91% at the age of 18–24 months to 9.56% at the age of 66–71 months.

### All-cause mortality in the SCP

Among the 2 191 956 subjects of the birth cohort, 1135 subjects died during the first 12 months of life. We analyzed the mortality for subjects alive 1 year after birth (n = 2 190 821). 1414 deaths were recorded in the GP and 91 deaths in the SCP during the follow-up until October 2016. All-cause mortality was 0.09 deaths/1000 person-years (95% CI, 0.08–0.09) in the subjects in the GP and 2.85 deaths/1000 person-years (95% CI, 2.32–3.50) in the SCP, indicating that incidence rate ratio for all-cause mortality was 32.15 (95% CI, 25.72–39.76) in the SCP (Table 2).

**Table 2:** Comparison of all-cause mortality^a^ after the age of 1 year with maximal 10-year follow-up.

	Subjects in general population			Subjects with cerebral palsy			Incidence rate ratio^b^ for all-cause mortality (95% CI)
Gender	Person-years	Number of deaths	Mortality (95% CI)	Person-years	Number of deaths	Mortality (95% CI)	
Boys	8 215 627	826	0.10 (0.09–0.11)	18 516	40	2.16 (1.58–2.95)	21.49 (15.24–29.51)
Girls	7 736 445	588	0.08 (0.07–0.08)	13 415	51	3.80 (2.89–5.00)	50.02 (36.81–66.65)
Total	15 952 072	1414	0.09 (0.08–0.09)	31 931	91	2.85 (2.32–3.50)	32.15 (25.72–39.76)

CI, Confidence interval

^a^Number of deaths after the age of 1 year until October 2016 /1000 person-years

^b^Mortality of the subjects with cerebral palsy/ mortality of the subjects in the general population

Growth parameters as mortality risk factors

Table 3 shows the adjusted HR of death after the age of 1 year by Cox proportional hazard regression analysis. Presence of CP showed significantly higher adjusted HR of death at all NHSIC screenings after the age of 1 year (*p* < 0.001), where adjusted hazard ratio of death by presence of CP was 11.64 (95% CI, 6.32–21.44) at the age of 18–24 months, 11.27 (95% CI, 5.92–21.46) at the age of 30–36 months, 15.38 (95% CI, 8.44–28.02) at the age of 42–48 months, 18.03 (95% CI, 9.13–35.62) at the age of 54–60 months, and 24.85 (95% CI, 9.97–61.91) at the age of 66–71 months. In terms of gender, girls showed significantly lower adjusted HR (range, 0.56–0.70) of death at all NHSIC screenings after the age of 1 year (*p* < 0.001). The presence of underweight was significantly associated with higher mortality in both subjects with CP and GP (*p* < 0.001), where the adjusted hazard ratio of death was 2.60 (95% CI, 1.93–3.50) at the age of 18–24 months, 3.12 (95% CI, 2.25–4.35) at the age of 30–36 months, 4.37(95% CI, 3.05–6.25) at the age of 42–48 months, 5.12 (95% CI, 3.44–7.62) at the age of 54–60 months, and 4.17 (95% CI, 2.32–7.50) at the age of 66–71 months. Birth weight did not affect mortality in both subjects with CP and GP after the age of 1 year (*p* > 0.05). Therefore, while presence of CP shows higher mortality, underweight is an important growth parameter that affects all-cause mortality of the subjects in the GP and SCP.

**Table 3:** Adjusted hazard ratio (HR) of death after the age of 1 year by the age of each NHSIC screening.

Age at NHSIC screening (months)	Model 1 (18–24)		Model 2 (30–36)		Model 3 (42–48)		Model 4 (54–60)		Model 5 (66–71)	
Number of subjects	1 373 795		1 449 871		1 395 656		1 039 985		701 520	
Variables	HR (95% CI)	*p-value*	HR (95% CI)	*p-value*	HR (95% CI)	*p-value*	HR (95% CI)	*p-value*	HR (95% CI)	*p-value*
Cerebral palsy										
No	Ref		Ref		Ref		Ref		Ref	
Yes	11.64 (6.32–21.44)	<0.001	11.27 (5.92–21.46)	<0.001	15.38 (8.44–28.02)	<0.001	18.03 (9.13–35.62)	<0.001	24.85 (9.97–61.91)	<0.001
Gender										
Boy	Ref		Ref		Ref		Ref		Ref	
Girl	0.69 (0.59–0.81)	<0.001	0.70 (0.58–0.85)	<0.001	0.60 (0.48–0.76)	<0.001	0.59 (0.44–0.79)	<0.001	0.56 (0.37–0.86)	0.01
Birth year	0.94 (0.88–0.99)	0.03	0.94 (0.87–1.01)	0.07	0.97 (0.88–1.06)	0.47	097 (0.85–1.12)	0.72	1.16 (0.90–1.50)	0.26
Birth weight (g)										
≥2500	Ref		Ref		Ref		Ref		Ref	
<1000	2.28 (0.78–6.70)	0.13	2.37 (0.80–7.01)	0.12	0.93 (0.21–4.02)	0.92	0.50 (0.07–3.88)	0.51	-	-
1001–2499	1.17 (0.84–1.64)	0.36	1.40 (0.98–2.00)	0.06	1.22 (0.80–1.87)	0.36	0.98 (0.56–1.73)	0.95	0.74 (0.31–1.79)	0.50
Underweight										
No	Ref		Ref		Ref		Ref		Ref	
Yes	2.60 (1.93–3.50)	<0.001	3.12 (2.25–4.35)	<0.001	4.37 (3.05–6.25)	<0.001	5.12 (3.44–7.62)	0.00	4.17 (2.32–7.50)	<0.001

CI, confidence interval; -, No death was observed.; NHSIC, National Health Screening Program for Infants and Children; Ref, Reference

## Discussion

In this nationwide birth cohort, we found a CP prevalence rate of 2.0 per 1000 live births. The incidence rate ratio for all-cause mortality was 32.15 in SCP compared to the subjects in the GP. Underweight was the growth parameter that significantly affects all-cause mortality in both SCP and the subjects in the GP. Birth weight did not affect mortality in both SCP and the subjects in the GP after the age of 1 year (*p* > 0.05).

The overall prevalence of CP has been reported to range from 1.5 to 3.0 per 1000 live births [3,8,10,24–28], while the pooled overall prevalence of CP was 2.11 per 1000 live births as of 2013 [3]. A multi-site European population-based study from 1980–2003 reported a decreased prevalence in CP from 1.90 in 1980 to 1.77 in 2003 [9]. Our reported prevalence of 2.0 per 1000 live births in CP is compatible to other reports.

A study from Bangladesh reported that children with low BW had a significantly increased risk of becoming underweight, with a risk ratio of 1.47 [29]. Being underweight and low BW are not truly independent variables based on the current definition of underweight. A higher percentage of children with low BW would be at high risk of being underweight at follow-up assessments. A more accurate approach would be to determine criteria based on the rate of body weight gain over time. Therefore, we analyzed the rate of body weight gain as well as BW and underweight. Although the mechanism of association between low BW and underweight is beyond the scope of this study, bulbar involvement seen in CP could impose as an additional risk of follow-up underweight [12]. Children who are underweight may need gastrostomy, and in fact, gastrostomy placement and subsequent gastrostomy feeds may relieve the symptoms of underweight.

A previous report showed that about half of severely disabled CP children survived till the age of 20 [13]. The authors also suggest that BW and gestational age were less predictive of survival than functional disability [13]. We revealed that underweight affects all-cause mortality while BW does not. Underweight causes more major medical conditions and increased risk of death in both SCP and the subjects in the GP [7,12]. Whilst underweight had a strong effect on mortality, nevertheless one cannot exclude the impact of functional disability, which was not considered in the current manuscript.

The overall results of the current study must be interpreted with an appropriate degree of caution, since this study has several limitations as follows. First, while the deaths in the first year of life is highest in the pediatric population and the growth deceleration is most acute in the first year of life in children with CP [30], we were only able to identify the SCP after age of 1 year, since the most of diagnosis of CP was done around age of 1. Second, the birth cohort only represents 93.5% of the total live births from 2007–2011 in South Korea. Therefore, the current birth cohort might not truly represent the total population. Third, 5102 subjects (0.2%) of the current birth cohort came from the NHIS, since they did not participate in the NHSIC. Therefore, we could not know the exact BW nor the data on the follow-up body weight and the follow-up weight-for-age percentile for these subjects. Fourth, while children can participate in the NHSIC screenings up to a maximum of 7 times from the age of 4–71 months, not all children in the current birth cohort participated in all NHSIC screenings. Participation in the NHSIC program is relatively low in children who have already received in-depth medical attention for any particular reason at certain age. Therefore, we could not know the follow-up body weight and the follow-up weight-for-age percentile data of children if they were not included in NHSIC screening. Fifth, the main limitation was that possible residual confounders on mortality were not presented. The gross motor functional classification system is known as one of best indicators of prognosis and mortality of CP. There are growth charts for height, weight, and BMI for boys and girls with CP, stratified by gross motor functional classification system. Since the gross motor functional classification system was unavailable in the current study, we were not able to use growth charts for SCP [7]. A British study reported that the strongest predictor of mortality was the capability of sitting by 24 months with 44.4 of HR along with other predictors such as fixed spinal curve, bulbar involvement, gastrostomy, and BW [12]. Data on these predictors were unavailable in the current study. Lastly, we were not able to identify causes or risk factors for CP such as perinatal asphyxia. We also could not present data on gestational age, since there were many empty or out-of-range data recorded in the questionnaire filled out by parents. Term babies with small for gestational age might exhibit certain differences when compared to subjects with low BW who were born prematurely.

Even with these limitations, to the best of our knowledge, this is the first study on growth and mortality which simultaneously followed up both the subjects in the subjects in the GP and SCP. While there is very little Asian data on prevalence of CP, growth parameters, and mortality of SCP, this analysis provides some guidance as to the management of SCP.

## Conclusion

In a nationwide birth cohort, we found a CP prevalence rate of 2.0 per 1000 live births. While subjects with CP shows higher mortality, underweight is an important growth parameter that affects all-cause mortality of both subjects with CP and GP. This study urges increased awareness that subjects with CP who are underweight require special care.

## Supporting information

S1 Checklist RECORD checklist. (DOC)

S1 Table Operational definition of cerebral palsy and corresponding number of subjects with cerebral palsy. (DOC)

S2 Table Number of subjects who had diseases that masquerade cerebral palsy. (DOC)

S3 Table Characteristics of birth cohort of 2007–2011. (DOC)

S4 Table Comparison of the age at each National Health Screening Program for Infants and Children (NHSIC) screening between the subjects in the general population and subjects with cerebral palsy. (DOC)
