# Supplementary material for: Which growth parameters can affect mortality in cerebral palsy?
Source: PLoS One. 2019 Jun 14;14(6):e0218320. doi: 10.1371/journal.pone.0218320 (PMC6568421; doi:10.1371/journal.pone.0218320)
Supplement: S1 Table — (DOC) [file pone.0218320.s002.doc]

**S1 Table. Operational definition of cerebral palsy (CP) and corresponding number of subjects with CP.**

| **Type of operational definition of CP** | **Condition 1** |  | **Condition 2** |  | **Condition 3** |  | **Condition 4** | **Number of subjects with CP** |
| --- | --- | --- | --- | --- | --- | --- | --- | --- |
| 1 | ≥1 admission with G80^a^ | OR | ≥10 visits as an out-patient with G80 within consecutive 5 years | OR | Disability registration with brain lesion & ≥1 admission & ≥2 visits as an out-patient with G80 | OR | - | 4443 |
| 2 | ≥1 admission with G80 | OR | ≥10 visits as an out-patient with G80 within consecutive 5 years | OR | Disability registration with brain lesion & ≥1 admission & ≥3 visits as an out-patient with G80 | OR | - | 4408 |
| 3 | ≥1 admission with G80 | OR | ≥10 visits as an out-patient with G80 within consecutive 5 years | OR | Disability registration with brain lesion & ≥1 admission & ≥5 visits as an out-patient with G80 | OR | - | 4366 |
| 4 | ≥1 admission & ≥2 visits to clinic as an out-patient with G80 | OR | ≥10 visits as an out-patient with G80 within consecutive 5 years | OR | Disability registration with brain lesion & ≥1 admission & ≥2 visits as an out-patient with G80 | OR | - | 4241 |
| 5 | ≥1 admission & ≥3 visits to clinic as an out-patient with G80 | OR | ≥10 visits as an out-patient with G80 within consecutive 5 years | OR | Disability registration with brain lesion & ≥1 admission & ≥2 visits as an out-patient with G80 | OR | - | 4201 |
| 6 | ≥1 admission & ≥5 visits to clinic as an out-patient with G80 | OR | ≥10 visits as an out-patient with G80 within consecutive 5 years | OR | Disability registration with brain lesion & ≥1 admission & ≥2 visits as an out-patient with G80 | OR | - | 4127 |
| 7 | ≥1 admission & ≥2 visits to clinic as an out-patient with G80 | OR | ≥10 visits as an out-patient with G80 within consecutive 5 years | OR | Disability registration with brain lesion & ≥1 admission & ≥3 visits as an out-patient with G80 | OR | - | 4206 |
| 8 | ≥1 admission & ≥3 visits to clinic as an out-patient with G80 | OR | ≥10 visits as an out-patient with G80 within consecutive 5 years | OR | Disability registration with brain lesion & ≥1 admission & ≥3 visits as an out-patient with G80 | OR | - | 4166 |
| 9 | ≥1 admission & ≥5 visits to clinic as an out-patient with G80 | OR | ≥10 visits as an out-patient with G80 within consecutive 5 years | OR | Disability registration with brain lesion & ≥1 admission & ≥3 visits as an out-patient with G80 | OR | - | 4092 |
| 10 | ≥1 admission & ≥2 visits to clinic as an out-patient with G80 | OR | ≥10 visits as an out-patient with G80 within consecutive 5 years | OR | Disability registration with brain lesion & ≥1 admission & ≥5 visits as an out-patient with G80 | OR | - | 4164 |
| 11 | ≥1 admission & ≥3 visits to clinic as an out-patient with G80 | OR | ≥10 visits as an out-patient with G80 within consecutive 5 years | OR | Disability registration with brain lesion & ≥1 admission & ≥5 visits as an out-patient with G80 | OR | - | 4124 |
| 12 | ≥1 admission & ≥5 visits to clinic as an out-patient with G80 | OR | ≥10 visits as an out-patient with G80 within consecutive 5 years | OR | Disability registration with brain lesion & ≥1 admission & ≥5 visits as an out-patient with G80 | OR | - | 4050 |
| 13 | ≥1 admission & ≥2 visits to clinic as an out-patient with G80 | OR | ≥10 visits as an out-patient with G80 within consecutive 3 years | OR | Disability registration with brain lesion & ≥1 admission & ≥2 visits as an out-patient with G80 | OR | - | 4598 |
| 14 | ≥1 admission & ≥3 visits to clinic as an out-patient with G80 | OR | ≥10 visits as an out-patient with G80 within consecutive 3 years | OR | Disability registration with brain lesion & ≥1 admission & ≥3 visits as an out-patient with G80 | OR | - | 4525 |
| 15 | ≥1 admission & ≥5 visits to clinic as an out-patient with G80 | OR | ≥10 visits as an out-patient with G80 within consecutive 3 years | OR | Disability registration with brain lesion & ≥1 admission & ≥5 visits as an out-patient with G80 | OR | - | 4411 |
| 16 | ≥1 admission & ≥5 visits to clinic as an out-patient with G80 | OR | ≥10 visits as an out-patient with G80 within consecutive 3 years | OR | Disability registration with brain lesion & ≥ 1 admission & ≥2 visits as an out-patient with G80 | OR | Disability registration with brain lesion & ≥5 visits to clinic as an out-patient with G80 | 4376 |

^a^The code of CP by the Korean Classification of Disease.
