# Supplementary material for: Which growth parameters can affect mortality in cerebral palsy?
Source: PLoS One. 2019 Jun 14;14(6):e0218320. doi: 10.1371/journal.pone.0218320 (PMC6568421; doi:10.1371/journal.pone.0218320)
Supplement: S2 Table — (DOC) [file pone.0218320.s003.doc]

**S2 Table. Number of subjects who had diseases that masquerade cerebral palsy (*n* = 3 956)^a^.**

| **Disease** | **Codes of Korean classification of diseases** | **Codes of Korean rare intractable disease program** | **Number of subjects (%)** |
| --- | --- | --- | --- |
| Metabolic disorders | E70–E71 | V117 | 1431 (36.17) |
| Lesch-Nyhan syndrome | E79.1 | V221 | 2 (0.05) |
| Other porphyria | E80.2 | V118 | 0 (0.00) |
| Disorders of copper metabolism | E83.0 | V119 | 73 (1.85) |
| Disorders of iron metabolism | E83.1 | V255 | 3 (0.08) |
| Disorders of phosphorus metabolism and phosphatases | E83.3 | V189 | 142 (3.59) |
| Amyloidosis | E85 | V121 | 0 (0.00) |
| Down’s syndrome | Q90 | V159 | 746 (18.86) |
| Edwards’ syndrome and Patau’s syndrome | Q91 | V160 | 26 (0.66) |
| Deletion of short arm of chromosome 5 | Q93.4 | V205 | 28 (0.71) |
| Other deletions of part of an chromosome | Q93.5 | V217 | 157 (3.97) |
| Turner’s syndrome | Q96 | V021 | 129 (3.26) |
| Other sex chromosome abnormalities, male phenotype, NEC | Q98 | V218 | 39 (0.99) |
| Fragile X chromosome | Q99.2 | V245 | 22 (0.56) |
| Myasthenia gravis and other myoneural disorders/ Primary disorders of muscles | G70/G71 | V012 | 558 (14.11) |
| Periodic paralysis | G72.3 | V258 | 0 (0.00) |
| Lambert-Eaton syndrome | G73.1 | V259 | 0 (0.00) |
| Other specified congenital malformation syndromes affecting multiple systems | Q87 | V185 | 172 (4.35) |
| Systemic atrophies primarily affecting the central nervous system | G10–G13 | V123 | 135 (3.41) |
| Phakomatoses, NEC | Q85 | V156 | 369 (9.33) |
| Total |  |  | 4032^a^ (101.92) |

NEC, not elsewhere classifiable.

^a^The number of subjects were 3956 and seventy-six subjects had 2 diagnoses.
