# Supplementary material for: Which growth parameters can affect mortality in cerebral palsy?
Source: PLoS One. 2019 Jun 14;14(6):e0218320. doi: 10.1371/journal.pone.0218320 (PMC6568421; doi:10.1371/journal.pone.0218320)
Supplement: S3 Table — (DOC) [file pone.0218320.s004.doc]

**S3 Table. Characteristics of the birth cohort of 2007-2011^a^.**

| **Gender** | **Birth cohort of 2007–2011** | | | **Total number of live births of 2007–2011 in South Korea** | **Proportion of the birth cohort over total live births (%)** |
| --- | --- | --- | --- | --- | --- |
| **Birth year** | **Number of subjects from NHSIC (%)** | **Number of subjects from NHIS (%)** | **Total number (%)** |
| Boys |  |  |  |  |  |
| 2007 | 220 178 (99.7) | 734 (0.3) | 220 912 (100) | 253 999 | 87.0 |
| 2008 | 221 499 (99.7) | 558 (0.3) | 222 057 (100) | 240 119 | 92.5 |
| 2009 | 218 334 (99.8) | 465 (0.2) | 218 799 (100) | 229 351 | 95.4 |
| 2010 | 233 137 (99.8) | 422 (0.2) | 233 559 (100) | 242 901 | 96.2 |
| 2011 | 233 333 (99.8) | 467 (0.2) | 233 800 (100) | 242 121 | 96.6 |
| 2007–2011 | 1 126 481 (99.8) | 2 646 (0.2) | 1 129 127 (100) | 1 208 491 | 93.4 |
| Girls |  |  |  |  |  |
| 2007 | 207 445 (99.7) | 702 (0.3) | 208 147 (100) | 239 190 | 87.0 |
| 2008 | 208 402 (99.7) | 561 (0.3) | 208 963 (100) | 225 773 | 92.6 |
| 2009 | 205 206 (99.8) | 379 (0.2) | 205 585 (100) | 215 498 | 95.4 |
| 2010 | 218 587 (99.8) | 369 (0.2) | 218 956 (100) | 227 270 | 96.3 |
| 2011 | 220 733 (99.8) | 445 (0.2) | 221 178 (100) | 229 144 | 96.5 |
| 2007–2011 | 1 060 373 (99.8) | 2 456 (0.2) | 1 062 829 (100) | 1 136 875 | 93.5 |
| Total |  |  |  |  |  |
| 2007 | 427 623 (99.7) | 1 436 (0.3) | 429 059 (100) | 493 189 | 87.0 |
| 2008 | 429 901 (99.7) | 1 119 (0.3) | 431 020 (100) | 465 892 | 92.5 |
| 2009 | 423 540 (99.8) | 844 (0.2) | 424 384 (100) | 444 849 | 95.4 |
| 2010 | 451 724 (99.8) | 791 (0.2) | 452 515 (100) | 470 171 | 96.2 |
| 2011 | 454 066 (99.8) | 912 (0.2) | 454 978 (100) | 471 265 | 96.5 |
| 2007–2011 | 2 186 854 (99.8) | 5 102 (0.2) | 2 191 956 (100) | 2 345 366 | 93.5 |

NHIS: the National Health Insurance Service; NHSIC: the National Health Screening Program for Infants and Children

^a^As of year 2016, the number of children under the age of 9 in South Korea were 4 566 168. There are 24 237 immigrants and long term residents under the age of 9 from other countries in South Korea. Therefore, ethnically non-Korean children under the age of 9, corresponded to 0.53% of total number of children under the age of 9.
