# Supplementary material for: Which growth parameters can affect mortality in cerebral palsy?
Source: PLoS One. 2019 Jun 14;14(6):e0218320. doi: 10.1371/journal.pone.0218320 (PMC6568421; doi:10.1371/journal.pone.0218320)
Supplement: S4 Table — (DOC) [file pone.0218320.s005.doc]

**S4 Table. Comparison of the age at the National Health Screening Program for Infants and Children (NHSIC) screening between the subjects in general population and subjects with cerebral palsy.**

| **NHSIC screening (months)** | **Number of participants** | **Age at NHSIC screening (mean ± standard deviation; days)** | | ***p*-value** |
| --- | --- | --- | --- | --- |
|  |  | **Subjects in general population** | **Subjects with cerebral palsy** |  |
| 3rd (18-24) | 1 373 795 | 657.83 ± 68.70 | 668.01 ± 69.26 | < 0.001 |
| 4th (30-36) | 1 449 871 | 1023.56 ± 68.20 | 1026.40 ± 68.68 | 0.07 |
| 5th (42-48) | 1 395 656 | 1385.06 ± 69.53 | 1384.68 ± 70.86 | 0.80 |
| 6th (54-60) | 1 039 985 | 1750.17 ± 70.30 | 1750.47 ± 69.00 | 0.86 |
| 7th (66-71) | 701 520 | 2103.71 ± 61.86 | 2102.95 ± 62.11 | 0.67 |
